# Patterns, motivations, and healthcare experiences of medical cannabis use among gender and sexuality diverse people in Australia: findings from the Cannabis as Medicine Survey 2022 (CAMS-22)

**DOI:** 10.1186/s12954-026-01489-9

**Published:** 2026-07-03

**Authors:** Benjamin T. Trevitt, Sasha Bailey, Llewelyn Mills, Thomas Arkell, Iain S. McGregor, Nicholas Lintzeris

**Affiliations:** 1 Drug and Alcohol Liaison Clinic, Royal Melbourne Hospital, Parkville, VIC Australia; 2 The Centre for Youth Mental Health (Orygen), University of Melbourne, Melbourne, Australia; 3 Drug and Alcohol Services, New South Wales Health, Sydney, Australia; 4 Specialty of Addiction Medicine, Faculty of Medicine and Health, University of Sydney, Sydney, Australia; 5 Drug and Alcohol Clinical Research and Improvement Network (DACRIN), Sydney, NSW Australia; 6 Centre for Human Psychopharmacology, Swinburne University of Technology, Melbourne, Australia; 7 School of Psychology, Faculty of Science, The University of Sydney, Sydney, NSW Australia; 8 Lambert Initiative for Cannabinoid Therapeutics, Brain and Mind Centre, The University of Sydney, Sydney, Australia

**Keywords:** Medical cannabis, LGBTQA+ people, Health-care providers, Healthcare experiences

## Abstract

**Introduction:**

LGBTQA+ people are at greater risk of using cannabis at earlier ages, in greater frequencies and quantities and over longer periods of time compared with their cisgendered, heterosexual peers. Prescribed medicinal cannabis is becoming increasingly commonly utilised in Australia to treat a variety of conditions including pain, mental health diagnoses and sleep. There is currently little research looking into the motivations and experiences of LGBTQA+ people accessing medical cannabis (MC) through healthcare providers.

**Objectives:**

To explore differences in patterns, reasons for using, and experiences of accessing medicinal cannabis between LGBTQA+ people and their cisgender, heterosexual peers.

**Methods:**

We utilised data collected from an online anonymous cross-sectional survey of individuals (CAMS-22 survey), consisting of adult Australians who had utilised cannabis to treat a medical condition in the previous year. Participants were asked questions about their gender and sexuality, demographic characteristics, patterns and characteristics of MC use, conditions treated, and accessibility to and satisfaction of treatment.

**Results:**

Of the 3107 respondents included in these analyses, 2332 (77.3%) self-identified as heterosexual and 686 (22.7%) as sexually diverse. Over 95% of participants identified as cisgender (n = 2932), with only 148 (4.8%) identifying as trans. Sexually diverse participants commenced regular MC use at an earlier age, were more likely to identify a mental health condition and less likely to identify pain as their primary reason for MC consumption, and reported less satisfaction with treatment compared to heterosexuals—primarily around provision of information. Trans participants commenced MC use at an earlier age; were more likely to identify a mental health condition or sleep as their primary reason for MC use; and also reported less satisfaction with treatment than their cisgender counterparts for more interpersonal reasons.

**Conclusion:**

LGBTQA+ people use cannabis for medical purposes at significantly earlier ages and primarily to address mental health and substance use concerns. Future research should explore ways in which prescribers can specifically tailor support to meet all physical and mental health needs of LGBTQA+ people, (beyond simply prescribing MC), not only through treatments that they offer, but also through adjunct and complementary services.

## Background

There is strong evidence that gender and sexuality diverse (henceforth LGBTQA+) people are at greater risk of using cannabis at earlier ages [1–3], in greater frequencies [2, 4] and quantities [2, 5, 6], and over longer periods of time [2, 3], compared with their cisgender, heterosexual peers. Cisgender people are defined as having a gender congruent with their sex recorded at birth and heterosexual people as having sexual and romantic attractions exclusively with people of the ‘other’ gender. Recently, studies have emerged exploring how LGBTQA+ people use cannabis for medical purposes [2], however findings are scarce and mixed [2, 7].

Since November 2016, medical practitioners in Australia have been able to prescribe medicinal cannabinoids as unregistered medicines via Special Access and Authorised Prescriber Schemes [8, 9]. While guidelines exist, practitioners may prescribe for any condition if able to provide justification to the regulatory body based on available evidence and current practices [8, 9]. Most products have not been formally approved nor assessed by the TGA for efficacy, safety, or quality [9, 10]. As is stands, prescribed medical cannabis (MC) is typically used as an adjunct to standard treatments, offering small to modest benefits for chronic pain, spasticity, chemotherapy-induced nausea and vomiting, and refractory epilepsy [11]. Moreover, there is only a paucity of evidence on their value in treating mental disorders and other medical conditions [11].

Analyses of amalgamated data drawn from the 2015–2017 US National Surveys on Drug Use and Health for adults (*N* = 126,463) found that both gay/lesbian women and bisexual women were over three times more likely to report cannabis use for medical purposes, (which they defined as use recommended by a doctor or other health-care professional), over the past 12 months relative to heterosexual women, irrespective of sociodemographic covariates [7]. On the other hand, another study drawing on an online sample of US-based adults aged 18 to 64 (*N* = 4334)—of which 25.1% were LGBTQA+ (n = 1086)—found that LGBTQA+ people were less likely to use legal (prescribed or in a state where cannabis use without a prescription is legal) or illicit cannabis for medical only reasons (10.8% vs. 16.3%) and more likely to use legal/illicit cannabis for non-medical reasons (19.0% vs. 14.0%), relative to their cisgender, heterosexual peers [2].

Two consecutive US-based cross-sectional studies have found that trans people who use legal/illicit cannabis for either medical or recreational purposes were less likely to be categorised as daily cannabis consumers than their cisgender peers [2, 5] (41.5% vs 26.5%). However, they found that trans participants had a higher summed severity score of DSM-5 Cannabis Use Disorder (4.2 vs 3.4), suggesting that although trans people use cannabis less frequently they experience more negative consequences as a result of that use. In particular, Trans people were more likely to experience unsuccessful attempts at controlling/ceasing use (48.1% vs 32.5%); increased cravings (77.8% vs 66.4%) and more severe withdrawal symptoms (43.7% vs 27.9%); to fail to fulfil major obligations (13.3% vs 8.3%) and to give up or cut down important activities or hobbies (15.6% vs 7.3%) due to cannabis use. They were also more likely to continue cannabis use despite it causing/exacerbating physical or mental health problems (25.9% vs 17.3%) [5]. Detailed studies into patterns of medicinal cannabis use among LGBTQA+ people are required, with clearer delineation between differences due to gender and differences secondary to sexuality. This will enable a better understanding of the way patterns and experiences of use differ among these LGBTQA+ communities.

To date, much of the research into patterns of cannabis use for medical purposes among LGBTQA+ people has predominantly focused on MC use for alleviating mental health difficulties, including depressive [12] and post-traumatic traumatic stress symptoms [12, 13], and coping with stigma and discrimination [14–17]. Disparities in mental health between LGBTQA+ communities and their cisgender, heterosexual peers [18, 19] have been well documented and are often explained through the minority stress model [20–22]. This model posits that societal norms and assumptions among the majority of individuals that all people are cisgender and heterosexual breeds stigma and discrimination both internally within LGBTQA+ people (e.g., internalised feelings of shame about one’s gender and/or sexuality) and externally toward LGBTQA+ people (e.g., gender and sexuality-based bullying and victimisation) [22]. These experiences, often chronic and pervasive in all domains of daily life, cause mental ill-health, including depression, anxiety, and suicidality [22].

While it is important to research use of MC for mental health among LGBTQA+ people, there are very few studies examining other reasons for MC use among LGBTQA + people. A Canadian qualitative study exploring experiences of using cannabis among 50 young LGBTQA + men, noted that participants commonly discussed how cannabis was effective in treating insomnia, especially when ‘coming down’ from methamphetamines [12], and that cannabis was often used as a substitute for more harmful substances such as crystal methamphetamine and MDMA during chemsex practices to improve ability to consent and reduce risk of drug induced psychosis [8]. Within a mixed-methods study of 92 bisexual women in Canada, analysis of quantitative and qualitative responses found that many bisexual women within the sample reported that cannabis was effective in improving sleep (n = 48,52.2%), reducing pain (n = 33, 35.9%) and helping them relax (n = 77, 83.7%) [23]. Use of cannabis to treat sleep, pain, and mental health conditions is also common within the cisgender heterosexual population [24], however little research has focused on whether the way LGBTQA + people use MC differs significantly from cisgender heterosexual consumers.

There is little research examining experiences of LGBTQA + people accessing MC legally through healthcare providers: their satisfaction with the treatment offered, perceived quality of their medication, the side-effects they experience, the time it takes to access MC, and the cost. LGBTQA + people face significant barriers to accessing health services, including a lack of trust toward service providers targeting the general population alongside common occurrences of gender and sexuality-based discrimination [25]. In addition, trans people typically experience more difficulties accessing health services as many clinicians and other health service providers de-value and disrespect their genders [26]. Exploring the experiences of LGBTQA + people accessing prescribed MC is critical to help the system provide optimally LGBTQA + affirmative care.

Using data from the Cannabis as Medicine Survey 2022–2023 (CAMS-22) [24], this study aims to explore differences in (1) patterns, (2) reasons for using, and (3) experiences of accessing cannabis for medical purposes between LGBTQA + people, relative to their cisgender, heterosexual peers.

## Methods

The CAMS-22 survey [24] was an online anonymous cross-sectional survey of Australian adults who reported cannabis use for medical reasons within the previous 12 months. Study data was collected via Research Electronic Data Capture (REDCap), a secure web-based application, [24] from December 2022 to April 2023 [24].To be included in this study participants had to: i) give consent, (ii) be an Australian resident, (iii) 18 years-old or over, and (iv) have consumed MC in the previous year. We analysed their responses to survey questions examining: (i) baseline and demographic characteristics; (ii) patterns of MC use; (iii) primary reasons for MC use (where participants could only select one reason); (iv) general reasons for MC use (where participants could select multiple reasons) and (v) accessibility to and satisfaction with treatment. Participants were invited to participate online through social media and consumer group webpages, at conferences, as well as through private MC clinics [24].

Our focus in this analysis was to examine differences between sexuality and gender diverse participants and their heterosexual and cisgender counterparts. Only participants who were current MC users, defined as having used either illicit or prescribed medical cannabis in the previous 28 days, and who responded to questions related to gender and/or sexuality were included in this analysis. Sexuality diverse participants were defined as participants who did not identify as ‘Heterosexual’, and were grouped into the following categories: ‘Gay/Lesbian’; ‘Bisexual’; ‘Other’; ‘Prefer not to say’. To define gender, we asked participants about their sex assigned at birth (male or female) and their current gender identity (male; female; non-binary; different-term; prefer not to say). We also reviewed free-text responses provided by some participants regarding their gender identity. Trans people were participants who explicitly identified as non-binary or gender-diverse, or who indicated a current gender different from their assigned sex. Participants who responded ‘Prefer not to say’ to the above questions without providing further free-text responses were excluded from analyses. The relatively low number of trans people in the study (n = 148) meant that there were too few participants among the sub-groups of trans people (‘trans men’ (n = 21), ‘trans women’ (n = 18), ‘non-binary people’ (n = 87) and ‘gender-diverse people’ (n = 22) to make reliable estimates of differences between these sub-groups. For sensitivity analyses, we constructed two unadjusted bivariate models for each outcome: one with sexuality as a predictor and one with gender as a predictor to assess gender and sexuality-based differences in outcomes. Following this, we ran a series of adjusted models incorporating age, employment status, and highest level of education attained as model covariates. Initially, we undertook a series of preliminary adjusted models including gender (man, woman, and non-binary/gender diverse) as an additional covariate. However, due to small cell counts, (only four heterosexual participants were non-binary or gender diverse, and no cisgender people identified as non-binary), we elected to omit gender as a covariate.

We excluded all missing data from analysis, with no imputation. We used simple and multivariate regression models to analyse the data: Gaussian models were used to analyse continuous outcomes such as ‘Age’ and ‘Frequency of medical cannabis use’; Binary logistic regression models were used for binary categorical outcomes such as ‘Highest level of education’, ‘Employment’ and ‘Relationship status’’; Multinomial logistic regression models were used for multilevel categorical outcomes such as ‘Type of cannabis used’ and ‘Primary administration route of prescribed/illicit medicinal cannabis’; and Ordinal logistic regression models were utilised for outcomes related to treatment efficacy, stigmatisation and doctor-patient treatment satisfaction (e.g. ‘How satisfied were you with the amount of information provided by your doctor about possible benefits and side-effects’). The full questionnaire is provided in online supplementary materials. The Benjamini–Hochberg procedure was used to control the family-wise error rate. We performed the above statistical analyses using STATA v18. Only *p*-values < 0.05 were considered statistically significant.

## Results

Of the 5892 respondents who commenced the survey, 854 were initially excluded for either not providing consent (n = 325), not proceeding beyond the consent page (n = 522), not providing serious responses to questions (n = 5), or being test responses conducted by researchers (n = 2). Data were also excluded for 1344 participants who either did not indicate if they were currently consuming MC (n = 1208) or reported that they were not current MC users (n = 136). A small number of participants were also excluded from the study due to providing ambiguous responses to questions related to gender or sexuality (n = 3). Data were reported for the remaining 3107 respondents of whom 2845 (91.6%) completed the entire survey.

Most respondents self-identified as heterosexual (n = 2332, 77.3%); 686 (22.7%) were classified as sexually diverse with 185 (6.1%) identifying as gay or lesbian, 375 (12.4%) identifying as bisexual and 126 (4.2%) identifying as ‘other’. Over 95% of participants identified as cisgender (n = 2932, 95.2%), with only a minority identifying as trans (n = 148, 4.8%).

### Demographic outcomes

Table 1 shows differences between groups in demographic outcome variables and baseline characteristics. When grouped together, sexuality diverse participants were significantly younger (M_diff_ = 9.5 (8.4,10.6), *p* < 0.001), more likely to be Aboriginal or Torres Strait islander (OR 1.4 (1.0,2.0), *p* = 0.05), and more likely to have poorer baseline mental health (OR = 1.7 (1.44,2.02), *p* < 0.001) and sleep quality ratings (OR 1.9 (1.57,2.22), *p* < 0.001) and higher baseline fatigue levels (OR 1.4 (1.18,1.72), *p* < 0.001) than their heterosexual counterparts. They were also less likely to be currently in a relationship (OR 0.7 (0.6,0.8), *p* < 0.001). There were no significant differences in level of education or employment status, or baseline pain levels between the groups. Subgroup analyses based on sexuality revealed that gay/lesbian, bisexual and other (non-heterosexual) sexualities were all significantly younger than heterosexuals (M_diff_ = 7.1 (5.2,9.3), *p* < 0.001, M_diff_ = 10.4 (9.1,11.9), *p* < 0.001, and M_diff_ = 10.0 (7.7,11.3), *p* < 0.001 respectively). All subgroups were also significantly more likely to report poorer baseline mental health (OR 1.5 (1.11,1.99), *p* < 0.01, OR 1.8 (1.45,2.22), *p* < 0.001, OR 1.8 (1.26,2.46), *p* < 0.001) and sleep quality ratings (OR 1.6 (1.15,2.16), *p* < 0.01, OR = 1.3 (1.00,1.60), *p* = 0.05, OR 1.8 (1.20,2.62), *p* < 0.01)), and higher baseline fatigue levels (OR 1.5 (1.13,2.03), *p* < 0.01, OR 1.9 (1.52,2.34), *p* < 0.001, OR 2.5 (1.74,3.51), *p* < 0.001). Analyses based on gender indicated that trans participants were significantly younger (M_diff_ = 9.5 (8.4,10.6), *p* < 0.001) and less likely to have completed tertiary education than cisgender participants (OR 0.7 (0.5,1.0), *p* = 0.05), and more likely to have poorer baseline mental health (OR 1.6 (1.20,2.26), *p* < 0.01) and sleep quality ratings (OR 1.5 (1.01,2.20), *p* = 0.04) and higher baseline fatigue levels (OR 2.3 (1.63,3.12), *p* < 0.001). There were no significant differences between cis and trans participants in terms of Aboriginal, employment or relationship status, or baseline pain levels.

**Table 1:** Baseline characteristics of medical cannabis users based on sexuality and gender

Gender and Sexuality sub-groups	Heterosexual (n = 2332, 77.3%)		Gay/lesbian (n = 185, 6.1%)		Bisexual (n = 375, 12.4%)		Other (n = 126, 4.2%)		Sexually diverse (n = 686, 22.7%)		Cis (n = 2932, 95.2%)		Trans (n = 148, 4.8%)	
	M (SD)	Mdiff (SD)	M (SD)	Mdiff (SD)	M (SD)	Mdiff (SD)	M (SD)	Mdiff (SD)	M (SD)	Mdiff (SD)	M (SD)	Mdiff (SD)	M(SD)	Mdiff (SD)
Age, years	45.1 (13.2)	Ref	38.0 (11.8)	− 7.1(− 9.3,− 5.2)***	34.6 (11.4)	− 10.4(− 11.9,− 9.1) ***	35.1 (10.6)	− 10(− 12.3,− 7.7)***	35.6 (11.4)	− 9.5(− 10.6,− 8.4)***	43.6 (13.3)	Ref	32.1 (10.6)	− 11.5(− 13.7,− 9.4)***
Average pain level across last 7 days (out of 10)	6.1 (3.2)	Ref	5.6 (3.6)	**− **0.2(**− **0.72,0.22)	6.1 (3.1)	0.3(**− **0.03,0.67)	5.9 (3.0)	0.1(**− **0.49,0.65)	5.9 (3.2)	**− **0.1(**− **0.40,0.16)	6.1 (3.2)	Ref	5.9 (3.1)	0.0 (**− **0.48,0.57)

	N %	OR 95% CI	N %	OR 95% CI	N %	OR 95% CI	N %	OR 95% CI	N %	OR 95% CI	N %	OR 95% CI	N %	OR 95% CI
ATSI, Not ATSI [ref], n(%) ATSI	109/2332 (4.7)	Ref	14/185(7.6)	1.7(0.9,3.0)	24/375 (6.4)	1.4(0.9,2.2)	7/126 (5.6)	1.2(0.6,2.6)	45/686 (6.6)	**1.4 (1.0,2.0)**	144/2932(4.9)	Ref	12/148 (8.1)	1.7(0.9,3.2)
Highest level of education, (school [ref] vs tertiary, n(%) tertiary)	1807/2332 (77.5)	Ref	147/185 (79.5)	1.1(0.8,1.6)	289/375 (77.1)	1.0(0.8,1.3)	93/126 (73.8)	0.8(0.5,1.2)	529/686 (77.1)	1.0 (0.8,1.3)	2280/2932 (77.8)	Ref	104/148 (70.3)	0.7(0.5,1.0)*
Employment, (not**− **employed [ref] vs employed, n(%) employed)	1358/2332 (58.2)	Ref	117/185 (63.2)	1.2 (0.9,1.7)	211/375 (56.3)	0.9(0.7,1.2)	69/126 (54.8)	0.9(0.6,1.2)	397/686 (57.9)	1.0 (0.8,1.2)	1713/2932 (58.4)	Ref	75/148 (50.7)	0.7(0.5,1.0)
Relationship status, (single [ref] vs partnered, n(%) partnered)	1509/2332 (64.7)	Ref	98/185 (53.0)	0.6(0.5,0.8)**	225/375 (60.0)	0.8(0.7,1.0)	66/126 (52.4)	0.6(0.4,0.9)**	389/686 (56.7)	0.7(0.6,0.8)***	1845/2932 (62.9)	Ref	90/148 (60.8)	0.9(0.7,1.3)
Rating of MH over last.7 days^a^		Ref		1.5(1.11,1.99)**		1.8(1.45,2.22)***		1.8(1.26,2.46)***		1.7(1.44,2.02)****		Ref		1.6(1.20,2.26)**
Excellent	253/2157 (11.7)		9/167 (5.4)		18/347 (5.2)		4/120 (3.3)		31/634 (4.9)		280/2709 (10.3)		9/139 (6.5)	
Very Good	560/2157 (26.0)		41/167 (24.6)		53/347 (15.3)		22/120 (18.3)		116/634(18.3)		672/2709 (24.8)		17/139 (12.2)	
Good	722/2157 (33.5)		49/167 (29.3)		120/347 (34.6)		41/120 (34.2)		210/634 (33.1)		909/2709 (33.6)		46/139 (33.1)	
Fair	477/2157 (22.1)		50/167 (29.9)		107/347 (30.9)		37/120 (30.8)		194/634 (30.6)		642/2709 (23.7)		44/139 (31.7)	
Poor	145/2157 (6.7)		18/167 (10.8)		49/347 (14.1)		16/120 (13.3)		83.634 (13.1)		206/2709 (7.6)		23/139 (16.6)	
Rating of fatigue level over last 7 days^a^		Ref		1.5(1.13,2.03)**		1.9(1.52,2.34)***		2.5(1.74,3.51)***		1.9(1.57,2.22)***		Ref		2.3(1.63,3.12)***
None	337/2157 (15.6)		20/167 (12.0)		22/347 (6.3)		10/120 (8.3)		52/634 (8.2)		387/2709 (14.3)		11/139 (7.9)	
Mild	967/2157 (44.8)		67/167 (40.1)		141/347 (40.6)		39/120 (32.5)		247/634 (39.0)		1196/2709 (44.2)		43/139 (30.9)	
Moderate	651/2157 (30.2)		55/167 (32.9)		132/347 (38.0)		43/120 (35.8)		230/634 (36.3)		847/2709 (31.3)		53/139 (38.1)	
Severe	171/2157 (7.9)		22/167 (13.2)		34/347 (9.8)		23/120 (19.2)		79/634 (12.5)		228/2709 (8.4)		26/139 (18.7)	
Very Severe	31/2157 (1.4)		3/167 (1.8)		18/347 (5.2)		5/120 (4.2)		26/634 (4.1)		51/2709 (1.9)		6/139 (4.3)	
Rating of sleep quality over last 28 days^a^		Ref		1.6(1.15,2.16)**		1.3(1.00,1.60)*		1.8(1.20,2.62)**		1.4(1.18,1.72)***		Ref		1.5(1.02,2.21)*
Very Good	312/1574 (19.8)		21/138 (15.2)		34/262 (13.0)		12/90 (13.3)		67/490 (13.7)		581/2215 (26.2)		17/103 (16.5)	
Fairly Good	801/1574 (50.9)		68/138 (49.3)		146/262 (55.7)		46/90 (51.1)		260/490 (53.1)		1036/2215 (46.8)		48/103 (46.6)	
Fairly Bad	384/1574 (24.4)		39/138 (28.3)		66/262 (25.2)		24/90 (26.7)		129/490 (26.3)		494/2215 (22.3)		29/103 (28.2)	
Very Bad	77/1574 (4.9)		10/138 (7.3)		16/262 (6.1)		8/90 (8.9)		34/490 (6.9)		104/2215 (4.7)		9/103 (8.7)	

*indicates *p* < 0.05; **indicates *p* < 0.01; ***indicates *p* < 0.001

^a^Adjusted for age, education and employment

### Patterns and characteristics of medical cannabis use

#### Sexuality

Differences in MC use among different sexualities are reported in Table 2. The odds of sexually diverse participants using prescribed MC (as opposed to illicit MC only) was significantly lower than that of their heterosexual counterparts (OR 0.6 (0.53,0.80), *p* < 0.001). Additionally, the age at which sexually diverse participants commenced consuming both non-medical and MC regularly was significantly lower (M_diff_ = 1.4 (0.34,2.37), *p* < 0.01 and M_diff_ = 7.6 (6.48,8.76), *p* < 0.001 respectively), as was the average weekly cost of their MC use (M_diff_ = 14.6 (5.47,23.69), *p* < 0.01). The odds of sexually diverse participants primarily using smoked or vaporised prescribed MC products as opposed to oral prescribed MC products were lower than non-sexually diverse participants (OR 0.7 (0.52,0.94), *p* = 0.02, OR 0.8 (0.61,1.00), *p* = 0.05). There were no significant differences between heterosexual and sexually diverse participants in terms of frequency of medical or non-medical cannabis use, the percentage of cannabis use that was medical (vs nonmedical), or the primary administration route of illicit MC products. Subgroup analyses based on sexuality revealed that gays/lesbians, bisexuals, and other sexualities were less likely to use prescribed MC (as opposed to illicit only MC) than heterosexuals (OR 0.7 (0.48,0.96), *p* = 0.03, OR 0.7 (0.50,0.84), *p* < 0.01, OR 0.6 (0.41,0.90), *p* = 0.01 respectively), and commenced regular MC use at an earlier age (M_diff_ = 5.3 (3.26,7.40), *p* < 0.001, M_diff_ = 8.4 (6.85,9.88), *p* < 0.001, M_diff_ = 8.6 (6.06,11.08), *p* < 0.001). The odds of gay/lesbian and other sexualities primarily using smoked prescribed MC products as opposed to oral prescribed MC products were lower than that of heterosexuals (OR 0.5 (0.29,0.84), *p* < 0.01, OR 0.5 (0.27,1.00), *p* = 0.05), although no significant differences between the primary route of prescribed MC administration between gays/lesbians and heterosexuals were noted. Bisexuals and other sexualities spent less money per week on MC than their heterosexual peers (M_diff_ = 11.9 (0.28,23.47), *p* = 0.05, M_diff_ = 26.3 (8.03,44.64), *p* < 0.01), although there was no significant difference in average weekly expenditure between gays/lesbians and heterosexuals.

**Table 2:** Pattern and characteristics of medical cannabis use among gender and sexuality diverse people who use cannabis for medical purposes in Australia

Gender and Sexuality sub-groups	Heterosexual		Gay/lesbian			Bisexual			Other			Sexually Diverse			Cis		Trans		
	N %	OR 95%CI	N %	uOR 95%CI	aOR 95% CI	N %	uOR 95%CI	aOR 95% CI	N %	uOR 95%CI	aOR 95% CI	N %	uOR 95%CI	aOR 95% CI	N %	OR 95%CI	N %	uOR 95%CI	aOR 95% CI
Legality of current medical cannabis (illicit only [ref], vs prescribed, n(%) prescribed)^a^	1825/2332 (78.3)	Ref	134/185 (72.4)	0.7 (0.52,1.02)	0.7(0.48,0.96)*	266/375 (70.9)	0.7 (0.53,0.87)**	0.7 (0.50,0.84)**	87/126 (69.1)	**0.6** (0.42,0.92)*	0.6 (0.41,0.90)*	487/686 (71.0)	0.7 (0.56,0.82)***	0.6 (0.53,0.80)***	2262/2932 (77.2)	Ref	106/148 (71.6)	0.7 (0.52,1.08)	0.8 (0.53,1.14)
Type of cannabis, CBD [ref], n(%)^a^																			
THC	1317/2083 (63.2)	Ref	99/156 (63.5)	0.8 (0.52,1.28)	0.7 (0.42,1.06)	194/312 (62.2)	0.9 (0.66,1.34)	0.7 (0.47,0.99) *	57/103 (55.3)	0.7 (0.39, 1.17)	0.5 (0.28,0.86)*	350/571 (61.1)	0.9 (0.65,1.11)	0.6 (0.48,0.85)**	1622/2594 (62.5)	Ref	82/123 (66.7)	1.0 (0.60, 1.70)	0.7 (0.41. 1.22)
THC-CBD	482/2083 (23.2)	Ref	31/156 (19.9)	0.7 (0.40, 1.19)	0.6 (0.37,1.10)	74/312 (23.7)	1.0 (0.66,1.46)	0.9 (0.57,1.31)	28/103 (17.5)	0.9 (0.49,1.67)	0.8 (0.42,1.47)	133/571 (23.3)	0.9 (0.65,1.20)	0.8 (0.56,1.08)	613/2594 (23.6)	Ref	23/123 (18.7)	0.7 (0.40,1.41)	0.7 (0.34,1.24)
Primary administration route of prescribed medicinal cannabis, oral [ref], n(%)																			
Smoked	419/1792 (23.4)	Ref	22/132 (16.7)	0.6 (0.38,1.06)	0.5 (0.29,0.84)**	73/259 (28.2)	1.3 (0.95,1.90)	0.9 (0.61,1.28)	16/84 (19.1)	0.8 (0.44,1.54)	0.5 (0.27,1.00)*	111/475 (23.4)	1.0 (0.78,1.35)	0.7 (0.52,0.94)*	514/2221 (23.1)	Ref	30/102 (29.4)	1.6 (0.92,2.67)	0.9 (0.54,1.66)
Vaporised	793/1792 (44.3)	Ref	62/132 (47.0)	0.9 (0.64,1.40)	0.8 (0.50,1.13)	111/259 (42.9)	1,1 (0.79,1.48)	0.8 (0.57,1.09)	41/84 (48.8)	1.1 (0.68,1.83)	0.8 (0.48,1.34)	214/475 (45.1)	1.0 (0.83,1.32)	0.8 (0.61,1.00)*	986/2221 (44.3)	Ref	45/102 (44.1)	1.2 (0.75,2.00)	0.9 (0.53,1.44)
Primary administration route of illicit medicinal cannabis, oral [ref], n(%)																			
Smoked	450/743 (60.6)	Ref	48/69 (69.6)	2.1 (0.95,4.44)	1.1 (0.49,2.51)	111/156 (71.2)	2.1 (1.24,3.58) **	1.0 (0.57,1.82)	32/48 (66.7)	1.4 (0.62,3.03)	0.5 (0.23,1.30)	191/273 (70.0)	1.9 (1.28,2.89)**	0.9 (0.59,1.47)	602/970 (62.1)	Ref	46/58 (79.3)	3.6 (1.28,10.10)*	1.5 (0.50, 4.39)
Vaporised	139/743 (18.7)	Ref	13/69 (18.8)	1.8 (0.72,4.47)	1.1 (0.43,2.83)	27/111 (17.3)	1.7 (0.88,3.15)	0.9 (0.46,1.80)	8/48 (16.7)	1.1 (0.40,3.03)	0.6 (0.19,1.60)	48/273 (17.6)	1.6 (0.95,2.57)	0.9 (0.51,1.49)	180/970 (18.6)	Ref	8/58 (13.8)	2.1 (0.62,7.06)	1.1 (0.30,3.74)

	M (SD)	Md (SD)	M (SD)	uMd (SD)	aMd (SD)	M (SD)	uMd (SD)	aMd (SD)	M (SD)	uMd (SD)	aMd (SD)	M (SD)	uMd (SD)	aMd (SD)	M (SD)	Md (SD)	M (SD)	uMd (SD)	aMd (SD)
Age of regular non-medical cannabis use, (years)^b^, (N=2374)	18.5 (10.8)	Ref	18.1 (11.1)	− 0.4 (− 2.20,1.37)	− 0.4 (− 2.18,1.39)	16.6 (9.8)	− **1.9 (**− **3.17,** − **0.61)**	− **1.9 (**− **3.19,** − **0.63)**	17.5 (11.3)	− 1.0 (− 3.15,1.21)	− 1.0 (− 3.22,1.14)	17.0 (10.4)	− **1.3 (**− **2.35, **− **0.32)**	− **1.4 (**− 2.37,0.34)**	18.2 (10.7)	Ref	17.6 (11.0)	− 0.6 (− 2.57,1.37)	− 0.7 (− 2.67,1.28)
Age of regular medical cannabis use, (years)^b^, (N=2985)	38.7 (14.7)	Ref	33.2 (12.3)	− **5.5 (**− **7.61,** − **3.37)**	− **5.3 (**− **7.40,** − **3.26)**	30.4 (11.2)	− **8.3 (**− **9.82,** − **6.70)**	− **8.4 (**− **9.88,** − **6.85)**	30.1 (11.3)	− **8.6 (**− **11.15,** − 6.01)***	− **8.6 (**− **11.08,** − 6.06)***	31.1 (11.6)	− **7.6 (**− **8.78,** − 6.34)***	− **7.6 (**− **8.76,** − 6.48)***	37.6 (14.4)	Ref	27.7 (10.7)	− **9.9 (**− **12.25,** − 7.49)***	− **10.0 (**− **12.30,** − 7.65)***
Frequency of medical cannabis use, days used in past 28 days (days)^a^ (N=3107)	23.5 (7.7)	Ref	22.7 (7.9)	− 0.8 (− 1.93,0.37)	− 0.8 (− 1.93,0.37)	23.2 (7.8)	− 0.3 (− 1.15,0,52)	0.1 (− 0.76,0.97)	22.9 (7.7)	− 0.6 (− 1.97,0.78)	− 0.3 (− 1.65,1.11)	23.0 (7.8)	− 0.5 (− 1.15,0.16)	− 0.1 (− 0.78,0.58)	23.5 (7.7)	Ref	23.0 (8.0)	− 0.5 (− 1.78,0.76)	− 0.2 (− 1.49,1.07)
Duration of regular medicinal cannabis use, (years)^a^(N=2985)	6.4 (9.9)	Ref	4.7 (8.6)	− **1.7 (**− **3.11,** − 0.26)*	− 0.4 (− 1.83,0.94)	4.2 (6.6)	− **2.2 (**− **3.25,** − 1.15) ***	− 0.6 (− 1.64,0.47)	4.9 (7.8)	− 1.4 (− 3.17,0.30)	0.1 (− 1.64,1.75)	4.4 (7.4)	− **1.9 (**− **2.74,** − 1.11)***	− 0.4 (− 1.26,0.40)	6.0 (9.6)	Ref	4.5 (7.1)	− 1.5 (− 3.11,0.56)	0.1 (− 1.48,1.64)
Frequency of non-medical cannabis use, days used in past 28 days (days)^a^ (N=2376)	4.6 (9.0)	Ref	4.9 (8.4)	0.3 (− 1.16,1.76)	0.3 (− 1.15,1.77)	4.1 (7.7)	− 0.5 (− 1.53,0.57)	− 0.5 (− 1.55,0.54)	5.1 (9.0)	0.5 (− 1.31,2.25)	0.4 (− 1.39,2.17)	4.5 (8.1)	− 0.1 (− 0.94,0.72)	− 0.1 (− 0.94,0.80)	4.5 (8.8)	Ref	4.9 (8.7)	0.3 (− 1.27,1.95)	0.3 (− 1.33,1.93)
Percentage of cannabis use that is medical^a^ (N=2376)	85.9 (20.2)	Ref	85.4 (19.1)	− 0.6 (− 3.87,2.75)	0.2 (− 3.10,3.53)	84.5 (18.7)	− 1.4 (− 3.75,1.00)	− 0.1 (− 2.58,2.33)	86.4 (18.7)	0.5 (− 3.52,4.56)	1.7 (− 2.35,5.79)	85.1 (18.7)	− 0.8 (− 2.71,1.05)	0.3 (− 1.68,0.80)	85.8 (19.9)	Ref	85.0 (18.9)	− 0.8 (− 4.44, 2.85)	0.6 (− 3.15, 4.26)
Average weekly cost of medical cannabis use^a,c^ (N=2886)	103.8 (101.7)	Ref	96.6 (88.4)	− 7.2 (− 22.77,8.40)	− 11.9 (− 27.28,3.45)	104.2 (101.6)	0.3 (− 11.09,11.78)	− **11.9 (**− **23.47,** − 0.29)*	90.5 (97.5)	− 13.4 (− 31.89,5.15)	− **26.3 (**− **44.63, **− 8.03)*	99.6 (97.4)	− 4.3 (− 13.17,4.61)	− **14.6 (**− **23.69,** − 5.47)**	103.8 (101.6)	Ref	85.8 (70.2)	− 18.0 (− 34.97, − 1.12)***	− 33.6 (− 50.46, − 16.80)***

uOR, unadjusted odds ratio; aOR, adjusted odds ratio; uMd, unadjusted mean difference; aMD, adjusted mean difference

*indicates *p* < 0.05; ** indicates *p* < 0.01; *** indicates *p* < 0.001

^a^Estimates and confidence intervals were adjusted for basic demographics (age, education and employment)

^b^Estimates and confidence intervals were adjusted for above basic demographics excluding age

^c^Those reporting zero dollars in weekly cost of medical cannabis use were excluded

#### Gender identity

Cis and trans participants also differed significantly in terms of age of commencement of regular MC use and average weekly cost of MC use. Trans participants initiated regular MC use at a significantly earlier age (M_diff_ = 10.0 (7.65,12.30), *p* < 0.001) but spent less money per week on MC (M_diff_ = 33.6 (16.80, 50.46), *p* < 0.001) than their cis counterparts. There were no significant differences between cis and trans participants in odds of using prescribed MC (versus illicit-only use), composition of MC used, primary administration route of prescribed or illicit MC, age of regular non-medical cannabis use, frequency of medical or non-medical cannabis use, or percentage of cannabis use that was medical. For complete details, see Table 2.

### Primary health conditions treated with medical cannabis

#### Sexuality

The primary health conditions for which participants used MC, stratified by sexuality, are summarised in Table 3. Mental health and substance use was the primary condition most commonly treated with MC among sexuality diverse participants (n = 321, 47.1%) versus pain for heterosexual participants (n = 897, 38.8%). The odds of sexuality-diverse participants primarily using MC to treat a mental health condition were higher than those of heterosexual participants (OR 1.3 (1.08,1.56), *p* < 0.01), however there was no significant difference in either group’s odds of using MC to treat pain. With regard to specific conditions, the odds of sexually diverse people identifying anxiety, PTSD or fibromyalgia as their primary reason for MC use were significantly higher than that of their heterosexual counterparts (OR 1.2 (1.00,1.53), *p* = 0.05, OR 2.1 (1.44,3.14), *p* < 0.001, OR 2.8 (1.73,4.54), *p* < 0.001 respectively) whilst the odds of them identifying backpain was significantly lower (OR 0.6 (0.43,0.80), *p* < 0.01). Subgroup analyses revealed that gays/lesbians had higher odds of using MC primarily for mental health and substance use (OR 1.6 (1.16,2.16), *p* < 0.01), anxiety (OR 1.7 (1.21,2.36), *p* < 0.01) and PTSD (OR 3.1 (1.82,5.37), *p* < 0.001), but lower odds of using MC primarily for pain (OR 0.6 (0.42,0.86), *p* < 0.01), compared to heterosexuals. Bisexuals had significantly higher odds of using MC primarily for fibromyalgia (OR 3.1 (1.76,5.49), *p* < 0.001) and PTSD (OR 2.2 (1.38,3.49), *p* < 0.01), but lower odds of using MC primarily for backpain (OR 0.5 (0.36,0.83), *p* < 0.01), compared to heterosexuals. Those of other sexualities had significantly higher odds of using MC for fibromyalgia (OR 4.2 (1.94,9.00), *p* < 0.001) and mental health and substance use (OR 1.5 (1.03,2.17), *p* = 0.04) but lower odds of using MC for backpain (OR 0.2 (0.08,0.62), *p* < 0.01), compared to heterosexuals.

**Table 3:** Primary reasons for medical cannabis use based on gender and sexuality

Gender and Sexuality sub-groups	Heterosexual (n = 2312)		Gay/lesbian (n = 184)			Bisexual (n = 372)			Other (n = 125)			Sexually Diverse (n = 681)			Cis (n = 2904)		Trans (n = 148)		
	N %	OR 95%CI	N %	uOR 95%CI	aOR 95% CI	N %	uOR 95%CI	aOR 95% CI	N %	uOR 95%CI	aOR 95% CI	N %	uOR 95%CI	aOR 95% CI	N %	OR 95%CI	N %	uOR 95%CI	aOR 95% CI
Pain (total), No [ref] vs Yes, n(%) Yes	897 (38.8)	Ref	44 (23.9)	0.5 (0.35,0.71)*	0.6 (0.42,0.86)*	122 (32.8)	0.8 (0.61,0.97)*	1.0 (0.80,1.30)	36 (28.8)	0.6 (0.43,0.95)*	0.8 (0.56,1.25)	202 (29.7)	0.7 (0.56,0.80)**	0.9 (0.70,1.04)	1071 (36.9)	Ref	45 (30.4)	0.8 (0.53,1.09)	1.0 (0.72,1.51)
Back pain, No [ref] vs Yes, n(%) Yes	331 (14.3)	Ref	22 (12.0)	0.8 (0.52,1.29)	0.9 (0.57,1.44)	27 (7.3)	0.5 (0.31,0.71)**	0.5 (0.36,0.83)**	4 (3.2)	0.2 (0.07,0.54)*	0.2 (0.08,0.62)**	53 (7.8)	0.5 (0.37,0.69)**	0.6 (0.43,0.80)**	373 (12.8)	Ref	18 (12.2)	0.9 (0.57,1.57)	1.2 (0.71,2.00)
Arthritis, No [ref] vs Yes, n(%) Yes	192 (8.3)	Ref	6 (3.3)	0.4 (0.16,0.85)*	0.5 (0.22,1.20)	13 (3.5)	0.4 (0.23,0.71)*	0.7 (0.38,1.24)	8 (6.4)	0.8 (0.36,1.57)	1.3 (0.62,2.79)	27 (4.0)	0.5 (0.30,0.69)**	0.7 (0.48,1.13)	218 (7.5)	Ref	5 (3.4)	0.4 (0.18,1.07)	0.9 (0.34,2.14)
Neuropathy, No [ref] vs Yes, n(%) Yes	78 (3.4)	Ref	6 (3.3)	1.0 (0.42,2.25)	1.1 (0.46,2.55)	11 (3.0)	0.9 (0.46,1.66)	1.0 (0.50,1.91)	3 (2.4)	0.7 (0.22,2.26)	0.8 (0.24,2.57)	20 (2.9)	0.9 (0.53,1.43)	1.0 (0.58,1.65)	9.6 (3.3)	Ref	2 (1.4)	0.4 (0.10,1.70)	0.4 (0.11,1.86)
Fibromyalgia, No [ref] vs Yes, n(%) Yes	54 (2.3)	Ref	5 (2.7)	1.2 (0.46,2.97)	1.5 (0.58,3.84)	20 (5.4)	2.4 (1.41,4.02)***	3.1 (1.76,5.49)***	9 (7.2)	**3.2 (1.56,6.73)**	4.2 (1.94,9.00)***	34 (5.0)	2.2 (1.42,3.41)***	2.8(1.73,4.54)***	80 (2.8)	Ref	8 (5.4)	2.0 (0.97,4.30)	2.3 (1.03,4.95)*
Headaches, No [ref] Vs Yes, n(%) Yes	35 (1.5)	Ref	1 (0.5)	0.4 (0.05,2.62)	0.3 (0.04,2.40)	13 (3.5)	2.4 (1.24,4.50)**	2.1 (1.04,4.13)	3 (2.4)	1.6 (0.49,5.28)	1.4 (0.43,4.83)	17 (2.5)	1.7 (0.93,3.00)	1.5 (0.79,2.73)	53 (1.8)	Ref	2 (1.4)	0.7 (0.18,3.08)	0.6 (0.15,2.61)
MHSU (total), No [ref] vs Yes, n(%) Yes	756 (32.7)	Ref	91 (49.5)	2.0 (1.49,2.73)**	1.6 (1.16,2.16)**	166 (44.6)	1.7 (1.33, 2.07)**	1.1 (0.88,1.41)	64 (51.2)	2.2 (1.50,3.08)*	1.5 (1.03,2.17)*	321 (47.1)	1.8 (1.54,2.18)**	1.3 (1.08,1.56)**	1026 (35.3)	Ref	68 (46.0)	1.6 (1.13,2.20)*	1.0 (0.70,1.41)
Anxiety, No [ref] vs Yes, n(%) Yes	424 (18.3)	Ref	57 (31.0)	2.0 (1.44,2.79)**	1.7 (1.21,2.36)**	81 (21.8)	1.2 (0.95,1.62)	1.0 (0.73,1.27)	37 (29.6)	1.9 (1.26,2.78)**	1.5 (0.99,2.24)	175 (25.7)	1.5 (1.26,1.88)*	1.2 (1.00,1.53)*	571 (19.7)	Ref	38 (25.7)	1.4 (0.98,2.09)	1.1 (0.73,1.61)
Depression, No [ref] vs Yes, n(%) Yes	108 (4.7)	Ref	6 (3.3)	0.7 (0.30,1.59)	0.5 (0.23,1.25)	24 (6.5)	1.4 (0.89,2.22)	1.0 (0.63,1.63)	11 (8.8)	2.0 (1.03,3,77)*	1.5 (0.76,2.87)	41 (6.0)	1.3 (0.90,1.90)	1.0 (0.65,1.43)	143 (4.9)	Ref	6 (4.1)	0.8 (0.36,1.90)	0.6(0.30,1.37)
PTSD, No [ref] vs Yes, n(%) Yes	84 (3.6)	Ref	19 (10.3)	3.1 (1.82,5.16)***	3.1 (1.82,5.37)***	30 (8.1)	2.3 (1.51,3.58)**	2.2 (1.38, 3.49)**	3 (2.4)	0.7 (0.20,2.09)	0.6 (0.19,1.96)	52 (7.6)	2.2 (1.54,3.14)***	2.1 (1.44,3.14)***	130 (4.5)	Ref	12 (8.1)	1.9 (1.03,3.52)*	1.6 (0.85,3.09)
Neurological disorder, No [ref] vs Yes, n(%) Yes	110 (4.8)	Ref	12 (6.5)	1.4 (0.76,2.59)	1.5 (0.79,2.78)	18 (4.8)	1.0 (0.61,1.70)	1.0 (0.60,1.74)	6 (4.8)	1.0 (0.44,2.34)	1.0 (0.42,2.34)	36 (5.3)	1.1 (0.76,1.65)	1.1 (0.75,1.72)	138 (4.8)	Ref	13 (8.8)	1.9 (1.08,3.53)*	1.9 (1.02,3.53)*
Sleep, No [ref] vs Yes, n(%) Yes	372 (16.1)	Ref	29 (15.8)	1.0 (0.65,1.48)	1.0 (0.64,1.48)	50 (13.4)	0.8 (0.59,1.11)	0.9 (0.62,1.21)	10 (8.0)	0.5 (0.24,0.87)*	**0.5 (0.25,0.94)**	89 (13.1)	0.8 (0.62,1.04)	0.8 (0.63,1.09)	457 (15.7)	Ref	13 (8.8)	**0.5 (0.29,0.93)**	0.6 (0.32,1.05)
Gastrointestinal disorder, No [ref] vs Yes, n(%) Yes	48 (2.1)	Ref	3 (1.6)	0.8 (0.24,2.54)	0.7 (0.22, 2.36)	6 (1.6)	0.8 (0.33,1.82)	0.7 (0.29,1.70)	5 (4.0)	2.0 (0.77,5.03)	1.8 (0.70,4.82)	14 (2.1)	0.9 (0.47,1.71)	0.9 (0.48,1.70)	64 (2.2)	Ref	4 (2.7)	1.2 (0.45,3.47)	1.2 (0.42,3.40)
Cancer, No [ref[ vs Yes, n(%) Yes	26 (1.1)	Ref	1 (0.5)	0.5 (0.07,3.57)	0.8 (0.10,5.95)	2 (0.5)	0.5 (0.11,2.01)	0.9 (0.21,4.01)	1 (0.8)	0.7 (0.10,5.27)	1.4 (0.18,10.42)	4 (0.6)	0.5 (0.14,1.54)	0.8 (0.22,2.78)	33 (1.1)	Ref	0 (0.0)	N/A	N/A
Other, No [ref] vs Yes, n(%) Yes	103 (4.5)	Ref	4 (2.2)	0.5 (0.17,1.31	0.6 (0.21,1.62)	8 (2.2)	0.5 (0.23,0.98)*	0.6 (0.29,1.31)	3 (2.4)	0.5 (0.17,1.69)	0.7 (0.21,2.29)	15 (2.2)	0.5 (0.27,0.86)*	0.6 (0.35,1.10)	115 (3.9)	Ref	5 (3.4)	0.9 (0.34,2.13)	1.2 (0.46,3.00)

*indicates *p* < 0.05; ** indicates *p* < 0.01; *** indicates *p* < 0.001

aOR adjusted odds ratios: estimates and confidence intervals were adjusted for basic demographics (age, education and employment)

#### Gender identity

The primary health conditions for which participants used MC, stratified by gender, are also summarised in Table 3. The odds of trans participants identifying mental health and substance use as the primary reason for using MC, (as well as for PTSD in particular), were significantly higher than that of cis participants (OR 1.6 (1.13,2.20), *p* < 0.01) (although this significance disappeared once adjusted for by age). On the other hand, the odds of cis and trans participants identifying pain as the primary reason for MC use were not significantly different. The odds of neurological disorders being identified as the primary reason for MC use were higher amongst trans participants than cis participants (OR 1.9 (1.02,3.53), *p* = 0.04) as was fibromyalgia (OR 2.3 (1.03,4.95), *p* = 0.04). There were no other significant differences.

### General reasons for medicinal cannabis use

#### Sexuality

General conditions for which participants used MC, stratified by sexuality, are summarised in Table 4. Mental health (n = 514, 74.9%) and sleep (n = 490, 71.4%) were the two most common general conditions treated with MC amongst sexuality diverse participants, whilst pain (n = 1563, 67.0%) and sleep (n = 1574, 67.5%) were the most common amongst heterosexual participants. Sexuality diverse participants were significantly more likely to identify mental health and substance use (OR 1.7 (1.43,2.14), *p* < 0.001), a neurological disorder (OR 1.4 (1.12,1.86), *p* < 0.01), and a gastrointestinal disorder (OR 1.4 (1.07,1.85), *p* = 0.01) as a reason for MC use than heterosexual participants. Subgroup analysis revealed that gays/lesbians and bisexuals had higher odds of using MC for mental health reasons (OR 1.6 (1.12,2.22), *p* < 0.001, OR 2.0 (1.50,2.57), *p* < 0.001 respectively); ‘others’ were more likely to use MC for neurological disorders (OR 2.5 (1.59,3.80). *p* < 0.001); and ‘bisexuals’ were more likely to use MC for gastrointestinal orders (OR 1.4 (1.01,2.04), *p* = 0.05) than heterosexuals. Sexually diverse participants were also significantly more likely to have identified a greater number of reasons for MC use than heterosexuals (OR 1.5 (1.25,1.72), *p* < 0.001). There were no other significant differences.

**Table 4:** Any Identified Reasons for medical cannabis use based on gender and sexuality

Gender and Sexuality sub-groups	Heterosexual (n = 2332)		Gay/lesbian (n = 185)			Bisexual (n = 375)			Other (n = 126)			Sexually diverse (n = 686)			Cis (n = 2932)		Trans (n = 148)		
	N %	OR 95%CI	N %	uOR 95%CI	aOR 95% CI	N %	uOR 95%CI	aOR 95% CI	N %	uOR 95%CI	aOR 95% CI	N %	uOR 95%CI	aOR 95% CI	N %	OR 95%CI	N %	uOR 95%CI	aOR 95% CI
Pain (total), No [ref] vs Yes, n(%) Yes	1563 (67.0)	Ref	80 (56.7)	0.6 (0.48,0.87)*	0.8 (0.56,103)	254 (67.7)	1.0 (0.82,1.30)	1.3 (0.99,1.62)	82 (65.1)	0.9 (0.63,1.34)	1.1 (0.75,1.61)	441 (64.3)	0.9 (0.74,1.06)	1.1 (0.9,1.28)	1954 (66.6)	Ref	93 (62.8)	0.8 (0.60,1.19)	1.0 (0.72,1.45)
MHSU (total), No [ref] vs Yes, n(%) Yes	1237 (53.0)	Ref	130 (70.3)	2.1 (1.51,2.90)***	1.6 (1.12,2.22)***	292 (77.9)	3.1 (2.41,4.03)***	2.0 (1.50,2.57)***	92 (73.0)	2.4 (1.60,3.58)***	1.5 (0.98,2.27)	514 (74.9)	2.6 (2.19,3.20)***	1.7 (1.42,2.14)***	1668 (56.9)	Ref	116 (78.4)	2.7 (1.84,4.09)***	1.5 (1.01,2.31)*
Sleep, No [ref] vs Yes, n(%) Yes	1574 (67.5)	Ref	138 (74.6)	1.4 (1.00,1.99)*	1.3 (0.95,1.89)	262 (69.9)	1.1 (0.88,1.42)	1.0 (0.80,1.32)	90 (71.4)	1.2 (0.81,1.79)	1.1 (0.74,1.65)	490 (71.4)	1.2 (1.00,1.45)*	1.1 (0.92,1.36)	2004 (68.4)	Ref	100 (67.6)	1.0 (0.68,1.37)	0.9 (0.59,1.22)
Neurological disorder, No [ref] vs Yes, n(%) Yes	282 (12.1)	Ref	25 (13.5)	1.1 (0.73,1.76)	1.2 (0.74,1.81)	59 (15.7)	1.4 (1.00,1.84)	1.3(0.93,1.78)	33 (26.2)	2.6 (1.70,3.91)***	2.5 (1.59,3.80)***	117 (17.1)	1.5 (1.18,1.89)**	1.4 (1.12,1.86)**	374 (12.8)	Ref	35 (23.7)	2.1 (1.43,3.14)***	1.9 (1.25,2.87)**
Gastrointestinal disorder, No [ref] vs Yes, n(%) Yes	229 (9.8)	Ref	22 (11.9)	1.2 (0.78,1.97)	1.2 (0.77,1.97)	52 (13.9)	1.5 (1.07,2.04)*	1.4 (1.01,2.00)*	20 (15.9)	1.7 (1.05,2.85)*	1.7 (1.00,2.75)	94 (13.7)	1.5 (1.13,1.88)*	1.4 (1.07,1.85)*	308 (10.5)	Ref	31 (21.0)	2.3 (1.49,3.41)***	2.1 (1.38,3.24)***
Cancer, No [ref[ vs Yes, n(%) Yes	111 (4.8)	Ref	4 (2.2)	0.4 (0.16,1.21)	0.7 (0.27,2.07)	6 (1.6)	0.3 (0.14,0.75)	0.6 (0.27,1.49)	2 (1.6)	0.3 (0.08,1.32)	0.6 (0.15,2.65)	12 (1.8)	0.4 (0.20,0.65)***	0.7 (0.36,1.25)	130 (4.4)	Ref	1 (0.7)	0.1 (0.02,1.06)	0.3 (0.04,2.28)
Other, No [ref] vs Yes, n(%) Yes	280 (12.0)	Ref	19 (10.3)	0.8 (0.51,1.37)	1.0 (0.58,1.58)	45 (12.0)	1.0 (0.71,1.40)	1.2 (0.82,1.67)	18 (14.3)	1.2 (0.73,2.04)	1.4 (0.83,2.38)	82 (12.0)	1.0 (0.77,1.29)	1.2 (0.87,1.52)	355 (12.1)	Ref	18 (12.2)	0.1 (0.61,1.67)	1.1 (0.68,1.91)
Total No. reasons		Ref		1.2 (0.94,1.59)	1.2 (0.90,1.54)		1.8 (1.44,2.14)***	1.6 (1.28,1.94)***		1.9 (1.34,2.64)***	1.7 (1.20,2.40)**		1.6 (1.37,1.87)***	1.5 (1.25,1.72)***		Ref		1.7 (1.28,2.37)***	1.4 (1.04,1.95)*
1	602 (25.8)		35 (18.9)			64 (17.1)			26 (20.6)			125 (18.2)			711 (24.3)		27 (18.2)		
2	840 (36.0)		74 (40.0)			114 (30.4)			35 (27.8)			223 (32.5)			1042 (35.5)		43 (29.1)		
3	618 (26.5)		53 (28.7)			126 (33.6)			31 (24.6)			210 (30.6)			806 (27.5)		42 (28.4)		
> = 4	272 (11.7)		23 (12.4)			71 (18.9)			34 (27.0)			128 (18.7)			373 (12.7)		36 (24.3)		

*indicates *p* < 0.05; ** indicates *p* < 0.01; *** indicates *p* < 0.001

aOR adjusted odds ratios: estimates and confidence intervals were adjusted for basic demographics (age, education and employment)

#### Gender identity

General conditions for which participants used MC, stratified by gender, are summarised in Table 4. Mental health (n = 100, 78.4%) and sleep (n = 100, 67.6%) were the two most common general conditions treated with MC amongst trans participants, whilst sleep (n = 2004, 68.4%) and pain (n = 1954, 66.6%) were the most common amongst cisgendered participants. Trans participants were significantly more likely to identify mental health and substance use (OR 1.5 (1.01,2.31), *p* = 0.05), neurological disorders (OR 1.9 (1.25,2.87), *p* < 0.01) and gastrointestinal disorders (OR 2.1 (1.38,3.24), *p* < 0.001) as a reason for MC use than cisgendered participants. Trans participants were also significantly more likely to have identified a greater number of reasons for MC use than heterosexuals (OR 1.4 (1.04,1.95), *p* = 0.03). There were no other significant differences.

### Accessibility to and satisfaction of treatment amongst sexuality diverse medical cannabis users

#### Sexuality

**Fig. 1:**
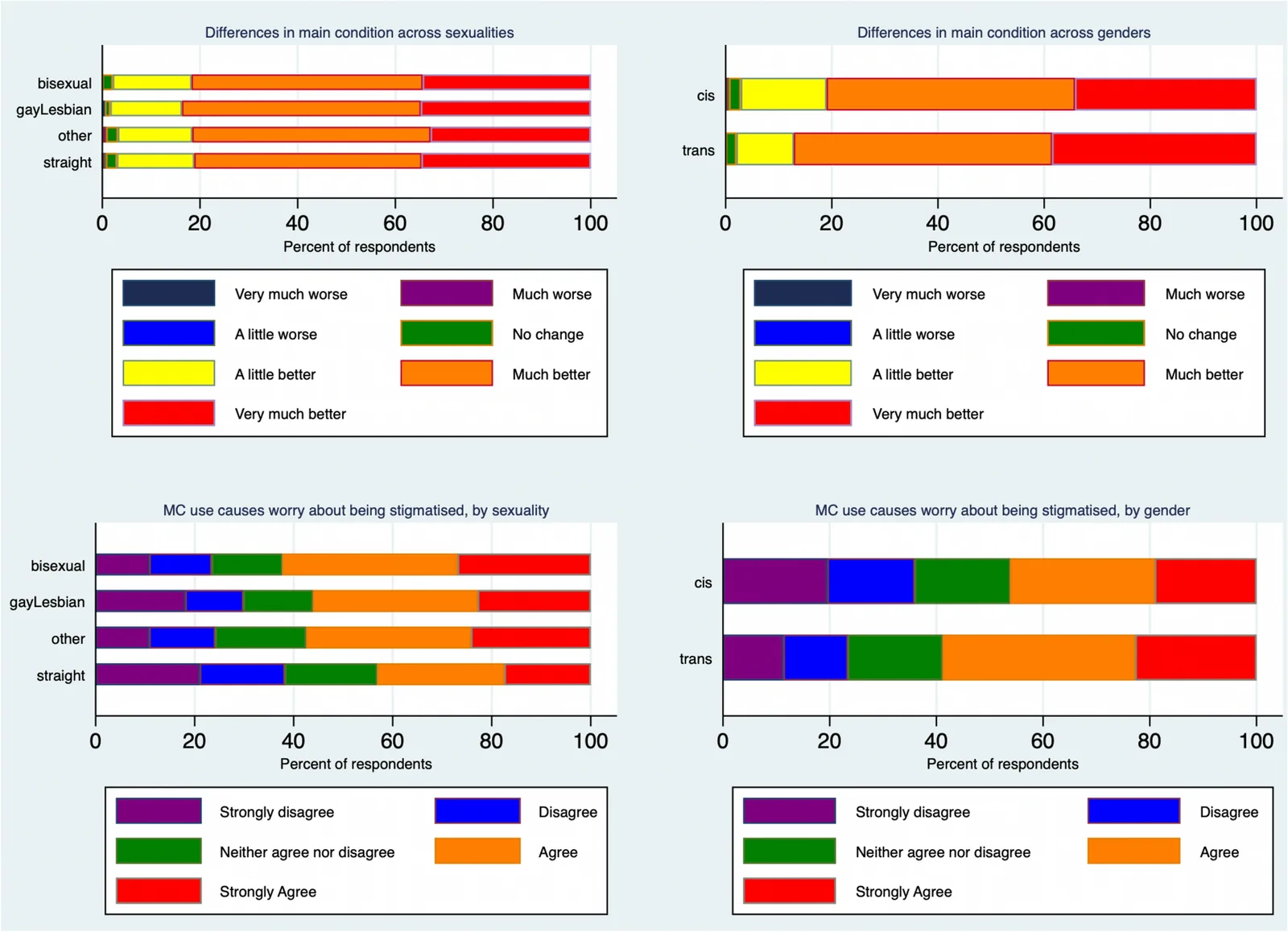
Patient reported impact of MC use

**Fig. 2:**
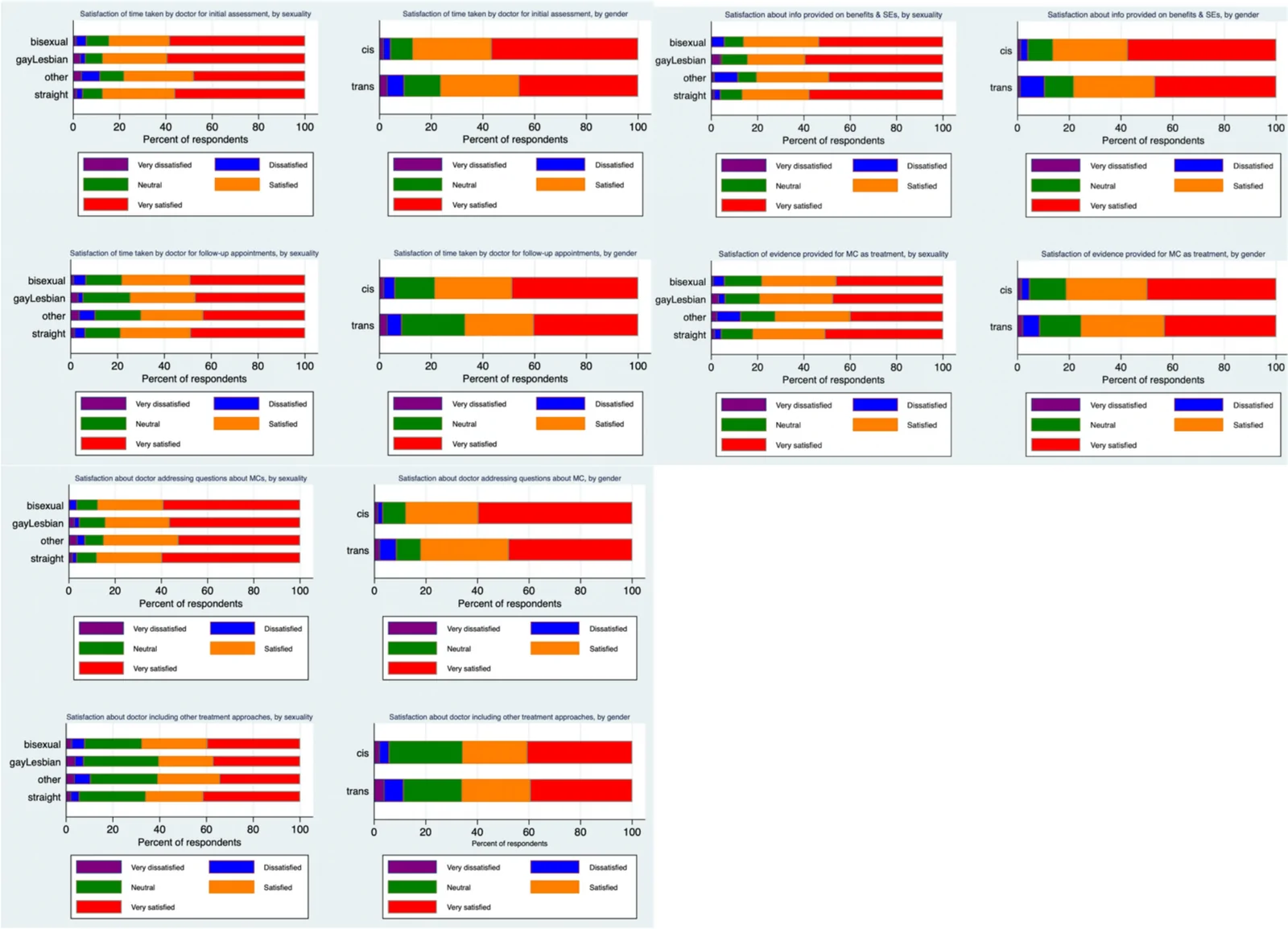
Patient reported satisfaction of MC treatment provision

The overwhelming majority of sexually diverse participants (n = 560, 82.2%) reported their main condition to be ‘much better’ or ‘very much better’ following MC initiation − with rates comparable to their heterosexual counterparts (n = 1877, 81.2%). However, sexually diverse participants as a whole reported being significantly more worried that their MC use would be stigmatised by medical professionals, friends or family than heterosexuals (OR 1.3 (1.13,1.57), *p* < 0.01). Additionally, whilst overall satisfaction rates were still positive, sexually diverse participants were significantly less satisfied with prescribed MC doctor-patient treatment than their heterosexual counterparts (M_diff_ = 0.5 (0.01,1.08), *p* = 0.05). Compared to heterosexual participants, sexually diverse participants were significantly less likely to be satisfied with the information provided by the doctor about benefits and side-effects (OR 0.8 (0.67, 1.00), *p* = 0.05); with the evidence provided for using MC as treatment for their condition (OR 0.8 (0.62,0.92), *p* = 0.05); with the way in which the doctor addressed their questions or concerns about MC (OR 0.8 (0.65,0.98), *p* = 0.03); and with the way in which the doctor included other approaches to treat their medical condition in addition to MC (OR 0.8 (0.64,0.96), *p* = 0.02). Please see Figs. 1 and 2 for an illustration of the above. Please see appendices Tables 5 and 6 for full tables of above.

#### Gender identity

Trans participants reported being significantly more worried that their MC use would be stigmatised by medical professionals, friends, or family than cisgendered participants (OR 1.6 (1.16, 2.09), *p* < 0.01). Additionally, trans participants were also significantly less satisfied with prescribed MC doctor-patient treatment as a whole (M_diff_ = 1.4 (0.39, 2.45), *p* < 0.01). Compared to cisgender participants, trans participants were significantly less likely to be satisfied with the information provided by their doctor about benefits and side-effects (OR 0.6 (0.40,0.86), *p* < 0.01), with amount of time taken by the doctor to do the initial (OR 0.5 (0.37,0.79), *p* < 0.01) or follow-up assessment (OR 0.6 (0.42,0.88), *p* < 0.01); and with the way in which the doctor addressed their questions or concerns about MC (OR 0.5 (0.38,0.80), *p* < 0.01). That said, the majority of trans participants reported being ‘satisfied’ or ‘very satisfied’ with the above criteria. Please see Figs. 1 and 2 for an illustration of the above (see appendices Tables 5 and 6 for full tables).

## Discussion

The aim of this study was to explore how the experiences of LGBTQA + people with MC differ from their heterosexual and cisgendered counterparts. On average, trans participants were 10 years younger when they commenced MC use than cisgendered participants whereas sexuality diverse participants were one year younger when they commenced MC younger than heterosexual participants. The few current studies in this area support our findings, suggesting that LGBQTA + people initiate cannabis use (for medical and/or recreational purposes) at significantly earlier ages [3, 4], and use cannabis significantly more frequently than cisgender heterosexual people [3, 4, 27, 28]. However, it is important to note the differing contexts of substance use for many LGBTQA + people as distinct from cisgender, heterosexual people.

Many LGBTQA + people, particularly trans young people, engage in substance use as a way to explore their gender and/or sexuality and to affirm LGBTQA + social and community connection. For this community, substance use can also often be used as a tool to cope with extreme situations and states of mind specific to LGBTQA + people, such as chronic emotional pain caused by gender dysphoria and the disproportionate levels of discrimination they experience from very young ages in homes and schools [28]. However, although cannabis use may provide LGBTQA + people with relief from this emotional pain, there is some evidence that cannabis use in adolescents and young adults is associated with the development of psychosis [29, 30], ADHD [29–31], depression [29, 32], and cognitive decline later in adulthood [29, 33, 34] One study focusing on LGBTQA + university students found cannabis use resulted in diminished motivation and emotional regulation and elevated signs of cannabis dependency [34, 35] In our study, there was no statistically significant difference between sexually diverse and heterosexual participants and cis and trans groups in terms of duration of regular MC consumption. This lack of a difference could be explained by the fact that both sexually diverse participants and trans participants were significantly younger than their heterosexual and cis counterparts and thus had actually been using MC for a shorter period of time. Recent data collected by the Australian Bureau of Statistics supports the fact that sexuality and gender diverse Australians (Age_Median_ = 30 years) tend to be younger than the rest of the population (Age_Median_ = 38 years) [36]. Thus, further research should strive to age-match trans and cis participants of all ages to better investigate the true differences between cis and trans individuals consuming MC.

We also found that, even after controlling for age, sexuality-diverse people were significantly more likely to identify mental health, particularly anxiety, depression and PTSD, as the primary (and a general) clinical condition for their MC use, relative to their heterosexual peers. This is understandable in the context of growing evidence of the disparities in mental health and substance use between LGBTQA + and heterosexual people [18, 37–41]. Studies in Australia and the United States estimate that approximately 60% of LGBTQA + youth experience high or very high levels of psychological distress [42] and that almost 70% undergo ‘persistent feelings of sadness or hopelessness’ [43], whilst only 30% of cisgender, heterosexual youths suffer the same degree of psychological distress [42] and only 29% report experiencing poor mental health [43]. Another report found that LGBTQA + adults are also more than twice as likely as cisgender, heterosexual adults to have a diagnosis of depression [44] and almost three times as likely to have experienced poor mental health within the preceding four weeks [44]. The underlying determinants driving these disparities are multi-faceted though are in part attributable to the chronic and often daily discrimination and identity-related minority stress faced by many members of the LGBTQA + community, including gender- and sexuality-based school bullying victimisation, parental rejection, and discrimination in workplace and health settings [38, 45]; as well as pains related to gender dysphoria for trans people in particular. There is currently limited evidence, (and the existing evidence is of poor quality), suggesting that MC use improves mental health for either the general population or LGBTQA + and it is a concern that so many in both groups are using cannabis for conditions for which there is no proven efficacy. Further research is thus required to determine if MC use for mental health reasons is a concern for LGBTQA + people, as well as the general population, who use MC and if it is, health-care providers should be trained to offer pharmacological treatment and counselling in a more holistic and LGBTQA + affirming way by providing a balanced LGBTQA + affirmative harm reduction approach.

Although mental health and substance use was the primary indication for MC use identified by LGBTQA + people, pain was the second most commonly identified primary reason, particularly amongst trans people. Our findings suggest that sexually diverse people and trans people are no more likely to identify pain as their primary (or a general) reason for MC use than their cisgendered heterosexual counterparts and that there is no difference between groups’ baseline pain levels. Interestingly, we found that sexually diverse and trans-participants were more likely to identify fibromyalgia as a specific primary reason for MC use than heterosexuals and cisgendered participants respectively. This is supported by other studies which have postulated that gender dysphoria and other psychological stressors could be potential contributing factors. Whilst other pain disparities amongst sexuality and gender diverse people and their cisgendered heterosexual counterparts have been previously noted in the literature [46–48], much of these disparities have been noted to have occurred as a result of experiences unique to the LGBTQA + community (e.g. pain associated with gender affirming hormonal therapies such as increased pelvic pain in trans men following initiation of testosterone [49] and increased headaches, breast and musculoskeletal pain in trans women following initiation of oestrogen and anti-androgen treatment [50, 51], as well as chronic post-operative pain following gender affirming surgical procedures in both trans men (e.g. mastectomies and masculinising facial surgeries) and trans women (e.g. facial feminisation surgeries and breast-augmentation)) [51].Unfortunately, our study did not explore in detail the types of pain specific to sexuality and gender diverse people. Additionally, our low sample size of some subgroups, (e.g. trans men (n = 21) and trans women (n = 18)), would have also made drawing any concrete conclusions challenging. Further research is required to ascertain the effectiveness of MC on specifically treating the unique types of pain experienced by LGBTQA + people, with additional concerted attention toward the role that MC may play in providing gender affirming care, (i.e. any single or combination of psychological, social, medical and/or surgical interventions provided to trans people with the aim to affirm and support their gender identity) [52].

Sleep was the third most common primary reason and second most common general reason for MC use identified by LGBTQA + community members. A narrative review assessing 31 papers concluded that sleep disturbances are more commonly reported by LGBTQA + adults compared to cisgender, heterosexual adults. [53]. This review found that both sexual minority adolescents and adults [53–58] and trans people [53, 59–62] were consistently more likely to have both poorer sleep quality and shorter sleep duration than cisgender heterosexual people; with bisexual women and trans people reporting the highest rates of sleep disturbance [53]. One study included in the review found that 60% of trans participants were consuming medications to address their sleep problems [53, 61]. Potential reasons behind these disparities include the higher rates of physical and mental health conditions faced by the LGBTQA + community [53], at least in part due to minority stressors, structural and interpersonal stigma [53, 63], discrimination [53, 63], and the disproportionate levels of anxiety, distress and gender dysphoria faced by the trans community [53, 61]. One study even found that administering oestrogen and anti-androgens to trans women resulted in an increase in duration of shallow sleep [53, 62] Our findings support the above in that sexually diverse people and trans people reported significantly higher levels of baseline fatigue and poorer sleep quality than heterosexual and cisgendered people. However interestingly, sexuality diverse people and trans people were no more likely to identify sleep as their primary or any reason for MC use than their cisgendered heterosexual counterparts. Potential reasons for this could be low sample size, especially in the trans group, that LGBTQ + groups were more likely to be already receiving alternative non-MC treatment for sleep disturbances prior to commencement of MC (e.g. other medication or therapy), or a failure of MC to effectively treat the underlying reasons for sleep disturbance specific to sexuality and gender diverse groups. Further research is required to ascertain the effectiveness of MC on specifically treating sleep disturbances and the underlying reasons for sleep disturbances experienced by sexuality and gender-diverse people.

LGBTQA + people were slightly more likely to report improvements in their main condition following initiation of MC. Conversely, LGBTQA + people were slightly more likely to report stigmatisation from family, friends and medical professionals regarding their MC use and have a slightly lower overall satisfaction with their clinician-patient MC treatment experiences compared to their cisgender, heterosexual counterparts. It is important to note that these differences while statistically significant were small, warranting caution in interpreting findings. Furthermore, the majority of participants in this study – irrespective of gender and sexuality – reported high levels of satisfaction across nearly all indicators measured. Whilst this finding of slightly reduced satisfaction among sexuality diverse participants may be explained by unmet needs for individualised and holistic information, findings of slightly higher dissatisfaction among trans participants may be interpreted as more experiential and interpersonal in nature, relating to time spent by the prescriber in the initial and follow-up appointments and the way in which the physician addressed their questions and concerns about MC.

Though we observed high rates of satisfaction with service provision across all indicators, our study highlights the importance of MC prescribers providing patient experiences that are accessible, LGBTQA + affirming, and knowledgeable about sexuality and gender diversity. LGBTQA + people often utilise online tools to assist with to contact health-care services and to identify professionals who are LGBTQA + themselves or an LGBTQA + ally, resulting in better longer-term engagement [64]. Further research should focus on delineating digital versus in-person service access as LGBTQA + people are more likely to access online services due to confidentiality and convenience, to avoid parents and friends finding out their gender and/or sexuality, and to minimise cost and transport barriers. In light of the potential connection between reasons for MC use among LGBTQA + people and their experiences of gender and sexuality, it is imperative that all healthcare providers, particularly those who regularly prescribe MC, to feel competent and capable of discussing LGBTQA + clients’ reasons for MC use in non-stigmatising, LGBTQA + affirmative way, providing MC options as well as other referrals to best address the clients’ needs. As they stand, MC-specific telehealth clinics almost certainly cannot be meeting these requirements [65]. In fact, there is growing concern Australia-wide of: some medical practitioners spending minimal time with patients before prescribing MC, with some consultations lasting as little as 4 min; some prescribing MC without even seeing the patient themselves (e.g. following a brief phone review with a nurse); and of some less experienced doctors being pressured to make poor decisions in prescribing MC (e.g. prescribing tetrahydrocannabinol (THC) when cannabidiol (CBD) might be more appropriate) or failing to consider other perhaps more appropriate options not involving MC [65]. Additionally, more than 85% of sexuality-diverse and trans participants reported that their MC prescriber was either not their regular GP or that they did not have a regular GP. Whether face-to-face or telehealth, to be able to successfully prescribe MC to an LGBTQA + client in a LGBTQA + affirming way requires a prescriber (ideally a GP) who is willing to also manage the client’s LGBTQA + specific physical and mental health needs holistically – which is clearly not being met under the current model of MC telehealth clinics. Furthermore, many healthcare providers both in Australia and internationally remain hesitant to prescribe MC due to the limited clinical evidence to support cannabis therapy [66, 67], lack of knowledge and support in MC prescribing [66–68], concerns over reputation and social stigma [66, 67], affordability [67], concerns about its appropriateness, and the potential, albeit rare, adverse events from MC use [67, 68]. Medical School Curricula and Continuing Medical Education (CME) and Mandatory Training programs need to be developed to support and encourage MC prescribers, (and the health workforce in Australia as a whole), and assist them to providing LGBTQA + specific and inclusive care to this population.

This study had several strengths, including a large national and diverse sample size; 22.7% of the participants identified as sexuality diverse and 4.8% identified as trans – the latter of which is in fact comparable to estimated prevalences world-wide [69, 70]. Other strengths include comprehensive measures of patterns of MC use and clinical indications, a systematic assessment of treatment access and satisfaction, and adequate gender and sexuality indicators that were collected contributing to the uniqueness of this MC survey. However, it also had several limitations. The fact it was an online survey may have led to selection bias in both the demographics of the people recruited (e.g. limited largely to people with regular access to the internet) as well as their experiences (people with more positive experiences related to MC may have been more likely to respond than those with more negative experiences)—thus generalisability to the Australian population of MC users at large is limited. Secondly, the survey did not include measures of sexual attraction or sexual behaviours and thus some sexuality diverse people may have been misclassified as heterosexual, also resulting in differential errors. Thirdly, gender items were also imperfectly collected using a two-question approach as opposed to the three-step approach (sex item, gender item permitting multiple selections if applicable from extensive list of suggested options with free-text response option, and gender item permitting one selection only from limited number of gender categories for analytic purposes) which is becoming increasingly cited as the gold-standard [71]. Fourthly, low prevalence rates in some of the sub-groups for specific primary conditions limit the interpretability of some results. Fifthly, several participants were unfortunately excluded from the study as they did not indicate if they were current MC users. Finally, there was a significant difference in age between trans and cis people included in the survey. Whether a true difference or perhaps due to selection bias, this also could affect the generalisability of results and the comparisons between the groups.

## Conclusion

This study reveals that LGBTQA + people, use cannabis for medical purposes at significantly earlier ages and more commonly to address concerns relating to mental health and substance use reasons, relative to their cisgender, heterosexual peers. In general, LGBTQA + people reported high levels of satisfaction among MC service provision across all indicators. Relatively lower levels of satisfaction (though still high, overall) with only a small minority of indicators relating to information supplied and the quality of care during MC treatment from primary healthcare providers, were observed. All healthcare providers should be knowledgeable of gender and sexuality diversity and those prescribing MC regularly should be particularly cognisant of how one’s gender and/or sexuality may impact one’s decisions to use MC. Future research should explore ways in which prescribers can specifically tailor support for LGBTQA + people using MC through adjunct and complementary services such digital-based harm reduction resources given higher propensity for LGBTQA + people to seek support online.

## Data Availability

The survey questions are available as an online supplement. Datasets will not be made publicly available, however data and code utilised in analyses can be requested from the corresponding author.

## Appendix

See Tables

**Table 5:** Efficacy, stigmatisation, and satisfaction of treatment amongst sexuality diverse medicinal cannabis users

Characteristic	Heterosexual (H)	Sexuality diverse (SD)	Gay/lesbian (GL)	Bisexual (BS)	Other (O)	Coefficient	Comparisons estimate (95% CI)	Adjusted estimate	Adjusted *p* values
Pharmaceutical efficacy									
Change in main condition following initiation of medicinal cannabis Very Much Worse Much Worse A Little Worse Neutral A Little Better Much Better Very Much Better	4 (0.2) 5 (0.2) 8 (0.4) 52 (2.3) 366 (15.8) 1076 (46.5) 801 (34.7)	1 (0.1) 1 (0.1) 0 (0.0) 13 (1.9) 106 (15.6) 327 (48.0) 233 (34.2)	1 (0.5) 0 (0.0) 0 (0.0) 2 (1.1) 27 (14.1) 90 (48.9) 64 (34.8)	0 (0.0) 0 (0.0) 0 (0.0) 8 (2.2) 60 (16.1) 176 (47.3) 128 (34.4)	0 (0.0) 1 (0.8) 0 (0.0) 3 (2.4) 19 (15.2) 61 (48.8) 41 (32.8)	Odds Ratio	SD-H: 1.0 (0.93,1.06) GL-H: 1.1 (0.81,1.41) BS-H: 1.0 (0.82,1.24) O–H: 1.0 (0.68,1.34)	SD-H: 1.0 (0.96,1.10) GL-H: 1.0 (0.74,1.30) BS-H: 0.9 (0.73,1.12) O–H: 0.9 (0.61,1.21)	0.36 0.88 0.60 0.60
Stigmatisation									
Medicinal cannabis use causes worry about being stigmatised by medical professionals, friends or family Strongly Disagree Disagree Neither agree or disagree Agree Strongly Agree	458 (21.1) 373 (17.1) 407 (18.7) 559 (25.7) 379 (17.4)	82 (12.8) 80 (12.5) 96 (15.0) 221 (34.5) 162 (25.3)	31 (18.1) 20 (11.7) 24 (14.0) 37 (33.3) 39 (22.8)	38 (10.9) 44 (12.6) 50 (14.3) 124 (35.4) 94 (26.9)	13 (10.8) 16 (13.3) 22 (18.3) 40 (33.3) 29 (24.2)	Odds Ratio	**SD-H: 1.8 (1.53,2.11)** **GL-H: 1.5 (1.13,1.97)** **BS-H: 2.0 (1.62,2.43)** **O–H: 1.8 (1.27,2.42)**	**SD-H: 1.3 (1.13,1.57)** GL-H: 1.2 (0.87,1.53) **BS-H: 1.4 (1.16,1.77)** O–H: 1.3 (0.94,1.81)	**0.001** 0.31 **0.03** 0.17
Doctor–patient treatment satisfaction									
Total doctor-patient treatment satisfaction (scores 1–5), mean (SD); Overall sum /35 (mean, SD)	28.9 (5.1)	28.5 (5.5)	28.6 (5.6)	28.7 (5.1)	27.5(6.2)	Beta	SD-H: -0.5 (-0.97,0.07) GL-H: -0.3 (-1.22,0.60) BS-H: -0.2 (-0.86,0.48) **O–H: -1.5 (-2.57,-0.33)**	**SD-H:-0.5 (-1.08,-0.01)** GL-H: -0.4(-1.29,0.55) BS-H: -0.3(-0.99, 0.40) **O–H: -1.6 (-2.68,-0.42)**	**0.05** 0.43 0.43 **0.02**
The amount of info provided by doctor about possible benefits and side effects Very Dissatisfied Dissatisfied Neutral Satisfied Very Satisfied	20 (1.1) 52 (2.9) 172 (9.4) 524 (28.7) 1057 (58.0)	6 (1.2) 25 (5.1) 44 (9.0) 146 (30.0) 266 (54.6)	5 (3.7) 1 (0.8) 15 (11.2) 33 (24.6) 80 (59.7)	0 (0.0) 14 (5.9) 20 (8.4) 78 (32.6) 127 (53.1)	1 (1.2) 8 (9.8) 7 (8.5) 26 (31.7) 40 (48.8)	Odds Ratio	SD-H: 0.9 (0.71,1.04) GL-H: 1.0 (0.72,1.44) BS-H: 0.9 (0.67,1.10) O–H: 0.7 (0.44,1.01)	**SD-H: 0.8 (0.67,1.00)** GL-H: 1.0 (0.70,1.41) BS-H: 0.8 (0.63,1.06) **O–H: 0.6 (0.42,0.96)**	**0.05** 0.95 0.19 0.10
The amount of info provided to me by doctor about evidence for using medical cannabis to treat my health condition Very Dissatisfied Dissatisfied Neutral Satisfied Very Satisfied	24 (1.3) 54 (3.0) 249 (13.7) 565 (31.0) 932 (51.1)	8 (1.6) 26 (5.2) 76 (15.6) 155 (31.8) 222 (45.6)	4 (3.0) 4 (3.0) 20 (14.9) 42 (31.3) 64 (47.8)	2 (0.8) 13 (4.9) 43 (16.2) 85 (32.0) 123 (46.2)	2 (2.3) 9 (10.3) 13 (14.9) 28 (32.2) 35 (40.2)	Odds Ratio	**SD-H: 0.8 (0.64,0.94)** GL-H: 0.9 (0.61,1.19) BS-H: 0.8 (0.64,1.03) **O–H: 0.6 (0.37,0.85)**	**SD-H: 0.8 (0.62,0.92)** GL-H: 0.8 (0.60,1.17) BS-H: 0.8 (0.61,1.01**)** **O–H: 0.6 (0.38,0.86)**	**0.006** 0.30 0.08 **0.02**
Amount of time doctor took to do the initial assessment Very Dissatisfied Dissatisfied Neutral Satisfied Very Satisfied	24 (1.3) 49 (2.7) 157 (8.6) 566 (31.0) 1028 (56.4)	10 (2.1) 22 (4.5) 45 (9.2) 132 (27.1) 278 (57.1)	4 (3.0) 3 (2.2) 10 (7.5) 37 (27.6) 80 (59.7)	3 (1.1) 12 (4.5) 26 (9.8) 69 (25.9) 156 (58.7)	3 (3.5) 7 (8.1) 9 (10.3) 26 (29.9) 42 (48.3)	Odds Ratio	SD-H: 1.0 (0.77,1.17) GL-H: 1.1 (0.78,1.57) BS-H: 1.0 (0.80,1.33) **O–H: 0.6 (0.42,0.97)**	SD-H: 1.0 (0.79,1.17) GL-H: 1.1 (0.75,1.53) BS-H: 1.0 (0.74,1.25) **O–H: 0.6 (0.39,0.91)**	0.39 0.78 0.78 **0.05**
Amount of time the doctor takes in follow-up appts Very Dissatisfied Dissatisfied Neutral Satisfied Very Satisfied	28 (1.5) 83 (4.6) 275 (15.1) 544 (29.8) 894 (49.1)	10 (2.1) 23 (4.7) 85 (17.5) 137 (28.1) 232 (47.6)	4 (3.0) 3 (2.2) 27 (20.2) 37 (27.6) 63 (47.0)	3 (1.1) 14 (5.3) 41 (15.4) 77 (29.0) 131 (49.3)	3 (3.5) 6 (6.9) 17 (19.5) 23 (26.4) 38 (43.7)	Odds Ratio	SD-H: 0.9 (0.76,1.10) GL-H: 0.9 (0.64,1.23) BS-H: 1.0 (0.78,1.27) O–H: 0.7 (0.48,1.08)	SD-H: 0.9 (0.72,1.07) GL-H: 0.9 (0.62,1.21) BS-H: 1.0 (0.74,1.23) O–H: 0.7 (0.46,1.04)	0.20 0.60 0.72 0.24
Doctor addresses questions or concerns about medical cannabis Very Dissatisfied Dissatisfied Neutral Satisfied Very Satisfied	25 (1.4) 36 (2.0) 158 (8.7) 510 (28.0) 1095 (60.0)	6 (1.2) 15 (3.1) 46 (9.5) 140 (28.8) 280 (57.5)	3 (2.2) 3 (2.2) 15 (11.2) 37 (27.6) 76 (56.7)	0 (0.0) 9 (3.4) 24 (9.0) 75 (28.2) 158 (59.4)	3 (3.5) 3 (3.5) 7 (8.0) 28 (32.2) 46 (52.9)	Odds Ratio	SD-H: 0.9 (0.73,1.08) GL-H: 0.8 (0.60,1.19) BS-H: 1.0 (0.76,1.26) O–H: 0.7 (0.49,1.12)	**SD-H: 0.8 (0.65,0.98)** GL-H: 0.8 (0.55,1.10) BS-H: 0.9 (0.66,1.13) O–H: 0.7 (0.43,1.01)	**0.03** 0.24 0.29 0.16
Doctor includes other approaches to treat medical condition I use medical cannabis for Very Dissatisfied Dissatisfied Neutral Satisfied Very Satisfied	34 (1.9) 67 (3.7) 518 (28.4) 446 (24.5) 759 (41.6)	14 (2.9) 26 (5.3) 133 (27.3) 128 (26.3) 186 (38.2)	5 (3.7) 5 (3.7) 43 (32.1) 31 (23.1) 50 (37.3)	6 (2.3) 15 (5.6) 65 (24.4) 74 (27.8) 106 (39.9)	3 (3.5) 6 (6.9) 25 (28.7) 23 (26.4) 30 (34.5)	Odds Ratio	SD-H: 0.9 (0.72,1.06) GL-H: 0.8 (0.58,1.11) BS-H: 1.0 (0.76,1.22) O–H: 0.7 (0.50,1.09)	**SD-H: 0.8 (0.64,0.96)** GL-H: 0.8 (0.54,1.04) BS-H: 0.9 (0.68,1.10) **O–H: 0.7 (0.44,0.98)**	**0.02** 0.13 0.25 0.13
The fees my doctor charges Very Dissatisfied Dissatisfied Neutral Satisfied Very Satisfied	176 (9.7) 314 (17.2) 598 (32.8) 389 (21.3) 347 (19.0)	38 (7.8) 97 (19.9) 154 (31.6) 105 (21.6) 93 (19.1)	10 (7.5) 20 (14.9) 47 (35.1) 28 (20.9) 29 (21.6)	19 (7.1) 60 (22.6) 77 (29.0) 60 (22.6) 50 (18.8)	9 (10.3) 17 (19.5) 30 (34.5) 17 (19.5) 14 (16.1)	Odds Ratio	SD-H: 1.0 (0.84,1.20) GL-H: 1.2 (0.86,1.60) BS-H: 1.0 (0.79,1.25) O–H: 0.8 (0.58,1.23)	SD-H: 1.1 (0.89,1.29) GL-H: 1.2 (0.90,1.68) BS-H: 1.1 (0.83,1.34) O–H: 0.9 (0.61,1.32)	0.46 0.61 0.64 0.64
Prescribing Doctor also treats other health problems Yes No/Have no other health problems	236 (12.9) 1578 (87.1)	51 (10.5) 436 (89.5)	15 (11.2) 119 (88.8)	25 (9.4) 241(90.6)	11 (12.6) 76 (87.4)	Odds Ratio	SD-H: 0.8 (0.57,1.08) GL-H: 0.8 (0.49,1.48) BS-H: 0.7 (0.45,1.08) O–H: 1.0 (0.51,1.86)	SD-H: 0.9 (0.62,1.22) GL-H: 0.9 (0.53,1.63) BS-H: 0.8 (0.50,1.22) O–H: 1.1 (0.56,2.08)	0.43 0.83 0.83 0.83

^5^ and

**Table 6:** Efficacy, stigmatisation and satisfaction of treatment amongst gender diverse medicinal cannabis users

Characteristic	Cis (n = 2040)	Trans (n = 98)	Coefficient	Comparisons estimate (95% CI)	Adjusted estimate (95% CI)	Adjusted *p* values
Pharmaceutical Efficacy						
Change in main condition following initiation of medicinal cannabis Very Much Worse Much Worse A Little Worse Neutral A Little Better Much Better Very Much Better	5 (0.2) 6 (0.2) 8 (0.3) 64 (2.2) 469 (16.2) 1359 (46.8) 993 (34.2)	0 (0.0) 0 (0.0) 0 (0.0) 3 (2.0) 16 (10.8) 72 (48.7) 57 (38.5)	Odds Ratio	T-C: 1.3 (0.95,1.76)	T-C: 1.2 (0.87,1.64)	0.27
Stigmatisation						
Medicinal cannabis use causes worry about being stigmatised by medical professionals, friends or family Strongly Disagree Disagree Neither agree or disagree Agree Strongly Agree	535 (19.6) 447 (16.4) 489 (17.9) 742 (27.2) 520 (19.0)	16 (11.4) 17 (12.1) 25 (17.7) 51 (36.2) 32 (22.7)	Odds Ratio	**T-C: 1.6 (1.16,2.09)**	T-C: 1.1 (0.81,1.49)	0.54
Doctor-Patient treatment satisfaction						
Total treatment satisfaction (scores 1–5), mean (SD); Overall sum /35 (mean, SD)	28.9 (5.1)	27.6 (5.9)	Beta	**T-C: -1.3 (-2.34,-0.32)**	**T-C: -1.4 (-2.45,-0.39)**	**0.007**
The amount of info provided by doctor about possible benefits and side effects Very Dissatisfied Dissatisfied Neutral Satisfied Very Satisfied	25 (1.1) 65 (2.9) 217 (9.6) 651 (28.8) 1303 (57.6)	1 (0.9) 10 (9.4) 12 (11.3) 33 (31.1) 50 (47.2)	Odds Ratio	**T-C: 0.6 (0.42,0.89)**	**T-C: 0.6 (0.40,0.86)**	**0.006**
The amount of info provided to me by doctor about evidence for using medical cannabis to treat my health condition Very Dissatisfied Dissatisfied Neutral Satisfied Very Satisfied	31 (1.4) 73 (3.2) 320 (14.2) 704 (31.1) 1133 (50.1)	2 (1.9) 7 (6.6) 17 (16.0) 34 (32.1) 46 (43.4)	Odds Ratio	T-C: 0.7 (0.51,1.05)	T-C: 0.7 (0.50,1.06)	0.10
Amount of time doctor took to do the initial assessment Very Dissatisfied Dissatisfied Neutral Satisfied Very Satisfied	31 (1.4) 64 (2.8) 195 (8.6) 685 (30.3) 1286 (56.9)	3 (2.8) 7 (6.6) 15 (14.2) 32 (30.2) 49 (46.2)	Odds Ratio	**T-C: 0.6 (0.40,0.85)**	**T-C: 0.5 (0.37,0.79)**	**0.001**
Amount of time the doctor takes in follow-up appts Very Dissatisfied Dissatisfied Neutral Satisfied Very Satisfied	34 (1.5) 99 (4.4) 350 (15.5) 671 (29.7) 1107 (49.0)	3 (2.8) 6 (5.7) 26 (24.5) 28 (26.4) 43 (40.6)	Odds Ratio	**T-C: 0.6 (0.45,0.92)**	**T-C: 0.6 (0.42,0.88)**	**0.008**
Doctor addresses questions or concerns about medical cannabis Very Dissatisfied Dissatisfied Neutral Satisfied Very Satisfied	28 (1.2) 44 (2.0) 202 (8.9) 632 (28.0) 1355 (59.9)	2 (1.9) 7 (6.6) 10 (9.4) 36 (34.0) 51 (48.1)	Odds Ratio	**T-C: 0.6 (0.42,0.88)**	**T-C: 0.5 (0.38,0.80)**	**0.002**
Doctor includes other approaches to treat medical condition I use medical cannabis for Very Dissatisfied Dissatisfied Neutral Satisfied Very Satisfied	43 (1.9) 87 (3.9) 643 (28.4) 564 (24.9) 924 (40.9)	4 (3.8) 8 (7.6) 24 (22.6) 28 (26.4) 42 (39.6)	Odds Ratio	T-C: 0.9 (0.63,1.31)	T-C: 0.8 (0.57,1.20)	0.31
The fees my doctor charges Very Dissatisfied Dissatisfied Neutral Satisfied Very Satisfied	207 (9.2) 401(17.7) 739 (32.7) 486 (21.5) 428 (18.9)	10 (9.4) 24 (22.6) 32 (30.2) 19 (17.9) 21 (19.8)	Odds Ratio	T-C: 0.9 (0.62,1.26)	T-C: 0.9 (0.66,1.35)	0.74
Prescribing Doctor also treats other health problems Yes No/Have no other health problems	279 (12.3) 1982 (87.7)	13 (12.3) 93 (87.7)	Odds Ratio	T-C: 1.0 (0.55,1.80)	T-C: 1.1 (0.62,2.08)	0.69

^6^.

## Abbreviations

CAMS-22: Cannabis as Medicine Survey 2022–2023
MC: Medical cannabis
REDCap: Research Electronic Data Capture
THC: Tetarahydrocannabinol
CBD: Cannabidiol
